# Mechanistic Insights and Therapeutic Potential of Small Nucleolar RNA Host Genes in the Carcinogenesis of Hepatocellular Carcinoma

**DOI:** 10.1002/cam4.71460

**Published:** 2025-12-31

**Authors:** Jiajia Luo, Zhangxiu Liao, Shuangxia Zhang

**Affiliations:** ^1^ School of Pharmacy Youjiang Medical University for Nationalities Baise Guangxi P. R. China; ^2^ School of Basic Medical Sciences Youjiang Medical University for Nationalities Baise Guangxi P. R. China; ^3^ Guangxi Clinical Medical Research Center for Hepatobiliary Diseases Baise Guangxi P. R. China; ^4^ Guangxi Higher Education Engineering Research Center for Gastrointestinal Microecology and Health Baise Guangxi P. R. China; ^5^ Baise City Key Laboratory of Gastrointestinal Microecology and Health Baise Guangxi P. R. China; ^6^ Key Laboratory of Basic Research and Transformation of Tumor Immunity and Infectious Diseases Youjiang Medical University for Nationalities Baise Guangxi P. R. China

**Keywords:** carcinogenic mechanisms, hepatocellular carcinoma, LncRNA SNHGs, molecular biology

## Abstract

**Background:**

Hepatocellular carcinoma (HCC) is one of the major factors endangering human health due to its poor prognosis, resulting from difficulties in early diagnosis and lack of effective treatment measures. Long non‐coding RNAs (lncRNAs), which are RNA molecules that do not translate into proteins, play essential roles in various tumor malignancy mechanisms. Among these, small nucleolar host genes (SNHGs) represent a specific subgroup of lncRNAs that have recently been shown to be critically involved in the development of HCC.

**Objective:**

This review aims to summarize recent findings regarding the role of SNHGs in HCC carcinogenesis, and to clarify their mechanistic functions in cancer progression as well as their potential as diagnostic markers and therapeutic targets.

**Methods:**

A comprehensive literature search was performed in the PubMed database using the following keywords: “small nucleolar host gene”, and “hepatocellular carcinoma”, or “liver cancer”. Relevant literature was retrieved and screened to synthesize the latest research findings on the roles of SNHGs in HCC carcinogenesis. No restrictions were predefined on the publication time of the included literature, and studies focusing on the mechanistic insights and therapeutic potential of SNHGs in HCC were prioritized for inclusion in this narrative review.

**Results:**

Abnormal expression of SNHGs in HCC affects many facets of the disease, including tumor cell proliferation, stemness, invasion, migration, apoptosis, autophagy, ferroptosis (an iron‐dependent form of cell death), and pyroptosis (a highly inflammatory form of programmed cell death), as well as metabolic processes such as glycolysis, lipid and cholesterol metabolism. Furthermore, these expressions can alter the tumor microenvironment and contribute to resistance against sorafenib, a common treatment for HCC. Numerous studies have further revealed SNHGs promote the onset and progression of HCC through competing endogenous RNA (ceRNA, different RNA molecules compete for same microRNAs) network. Additionally, recent research shows that non‐ceRNA mechanisms, including small nucleolar RNA (snoRNA) mediated regulation, epigenetic regulation, and modulation of mRNA stability, also contribute to SNHGs' regulation of HCC development and progression.

**Conclusion:**

SNHGs play critical regulatory roles in HCC carcinogenesis and progression through both ceRNA and non‐ceRNA mechanisms. They hold potential as promising diagnostic markers and therapeutic targets for HCC, providing new insights for the clinical management of this disease.

Hepatocellular carcinoma (HCC) represents a significant global health concern due to its high incidence and mortality rates [1, 2]. Despite advancements in early diagnosis and treatment modalities, the 5‐year survival rate for HCC remains below 20% [3, 4]. This poor prognosis is primarily due to the complexity of its pathogenesis, the absence of effective biomarkers and therapeutic targets [5]. Therefore, identifying novel diagnostic markers and therapeutic targets is necessary to overcome the complex biological challenges for HCC.

Long non‐coding RNAs (lncRNAs) are defined as transcripts longer than 200 nucleotides that do not undergo translation into a protein. These molecules perform various roles, including acting as molecular signals, scaffolds, decoys, guides, and organizers of chromatin structure, as well as functioning as sponges for microRNAs (miRNAs) to regulate the expression of DNA, RNA, and proteins [6]. Emerging evidence suggests that lncRNAs may serve as promising biomarkers and therapeutic targets in malignant tumors, particularly HCC, due to their significant involvement in tumorigenesis and progression [7, 8].

Notably, among diverse lncRNAs, small nucleolar host genes (SNHGs) represent a unique subgroup characterized by their intronic sequences that encode small nucleolar RNAs (snoRNAs) [6]. The expression of SNHGs directly impacts the levels of corresponding snoRNAs within the cell. Consequently, SNHGs not only exhibit functions typical of other lncRNAs but also modulate the levels of snoRNAs within cells. This dual role affects cancer development [9]. A total of 32 SNHG family members (SNHG1‐32) have been identified; some of these play crucial roles in various biological processes, including gene expression regulation and cellular signaling pathways [10].

In liver cancer tissues, most SNHGs are found to be overexpressed. Upregulation of SNHGs expression correlates with shorter overall survival (OS), recurrence‐free survival (RFS), disease‐free survival (DFS), larger tumor size and number, poor histologic grade, lymphatic metastasis, vein invasion, advanced tumor stage, portal vein tumor thrombosis (PVTT), and elevated alpha‐fetoprotein (AFP) levels [11, 12]. Numerous studies have demonstrated that aberrant expression of SNHGs in HCC profoundly impacts various aspects of the disease, including tumor cell proliferation, invasion, migration, apoptosis, autophagy, as well as resistance to sorafenib, and interactions within the tumor microenvironment [12, 13, 14]. Consequently, the exploration of SNHGs has emerged as a promising avenue for understanding the molecular underpinnings of HCC. Elucidating the role of SNHGs in HCC is essential for clarifying the mechanisms underlying its carcinogenesis and for developing new therapeutic strategies. This article aims to review the research progress of SNHGs in HCC, focusing on their abnormal expression, functional roles, molecular mechanisms, and potential as diagnostic markers and therapeutic targets.

## 1 Abnormal Expression of SNHGs in HCC

**FIGURE 1:**
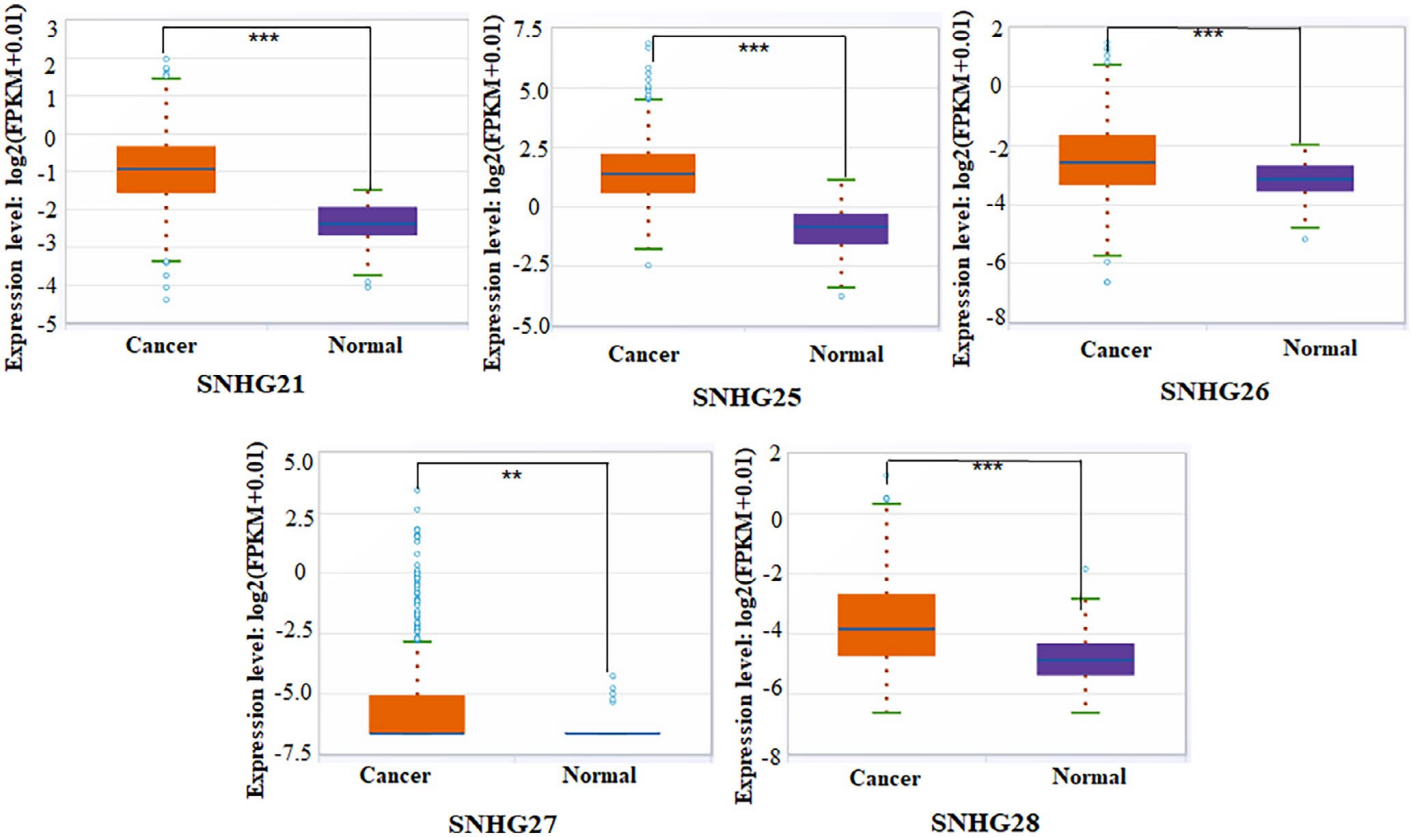
Expressions of SNHG21, SNHG25‐28 in hepatocellular carcinoma (HCC) tissues according to TCGA database by StarBase v3.0. As shown in the figure, the expression levels of SNHG21 and SNHG25‐28 in HCC tissues were significantly higher than those in the adjacent non‐tumor tissues. Compared with the normal group, ***p < 0.001, **p < 0.01.

Dysregulation of SNHGs has been increasingly recognized as a key factor in the pathogenesis of HCC. In HCC, the expression patterns of SNHGs are complex. Studies have shown that certain SNHGs, such as SNHG18, are downregulated in HCC tissues, while others, such as SNHG1, SNHG3‐17, and SNHG20‐23, show a marked increase in HCC tissues relative to normal liver tissues [12, 15]. However, studies on the expression of GAS5 (Growth Arrest‐Specific 5, also known as SNHG2) in HCC tissues have yielded inconsistent results. Some reports show differing expression patterns. For example, Wang C et al. reported low GAS5 expression in HCC cell lines and tissues. In contrast, data from the TCGA (The Cancer Genome Atlas) database and a study by Kim SY et al. involving Korean HCC patients showed high GAS5 expression [16, 17, 18]. Additionally, Eisa A reported that the expression of GAS5 correlates with patients' age, gender, ethnicity, and tumor stage in HCC [19]. Therefore, the inconsistency in GAS5 expression observed in HCC patients may be largely influenced by socio‐demographic factors such as age, gender, and ethnicity. To further explore gene expression patterns of several novel SNHGs in HCC, our group analyzed the expression levels of SNHG21 and SNHG25‐28 in liver cancer tissues from the TCGA database using the starBase platform (https://rnasysu.com/encori/panGeneDiffExp.php). We found that SNHG21 and SNHG25‐28 were significantly upregulated in HCC tissues compared to adjacent normal tissues (see Figure 1).

## 2 Impacts of Dysregulated SNHGs on the Biological Behavior of HCC Cells

The above studies indicate that the vast majority of SNHGs show abnormal expression in liver cancer tissues. These SNHGs may regulate various biological functions of liver cancer cells. Recent research reveals that SNHGs not only regulate biological behaviors such as proliferation, apoptosis, migration, and drug resistance but also influence other properties including stemness, ferroptosis, pyroptosis, and metabolic reprogramming in liver cancer cells [20, 21, 22, 23, 24]. Based on the reported results, we have constructed a table (Table 1) summarizing their regulatory impacts on the aforementioned biological behaviors of HCC cells.

**TABLE 1:** The Functional roles, related genes and pathways of SNHGs in HCC.

Functional roles	SNHG member	Interactions and related genes and pathways	Core validation systems
Accelerating the cell cycle	SNHG1	EZH2/CDKN1A and CDKN2B, miR‐140‐5p/CDK4	In vitro, in vivo, and clinical sample [25]
		BUB1, CCNA2, CCNB1, KIF11, NCAPG and TOP2A	In vitro and clinical sample [26]
		DNMT1/p53	In vitro and clinical sample [27]
	GAS5 (SNHG2)	miR‐423–3p/SMARCA4	In vitro, in vivo, and clinical sample [18]
	SNHG3	E2F1/NEIL3	In vitro and clinical sample [28]
	SNHG5	DNMT3a/SPATS2	In vitro [29]
		miR‐23c/HMGB2	In vitro and in vivo [30]
		miR‐26a‐5p/GSK‐3β	In vitro, in vivo, and clinical sample [31]
	SNHG6	miR‐204‐5p/E2F1	In vitro, in vivo, and clinical sample [32]
		miR‐139‐5p/SERPINH1	In vitro, in vivo, and clinical sample [33]
		miR‐101‐3p/ZEB1 and UPF1/TGFβ/Smad	In vitro, in vivo, and clinical sample [34]
		miR‐6509‐5p/HIF1A	In vitro, in vivo, and clinical sample [35]
	SNHG9	DNMT1, DNMT3A, and DNMT3B/GSTP1	In vitro and clinical sample [36]
	SNHG10	miR‐150‐5p/RPL4‐c‐Myb/SCARNA13	In vitro, in vivo, and clinical sample [37]
	SNHG12	miR‐516a‐5p/HEG1	In vitro and in vivo [38]
	DANCR (SNHG13)	miR‐216a‐5p/KLF12	In vitro and in vivo [39]
	SNHG15	miR‐18b‐5p/LMO4	In vitro, in vivo, and clinical sample [40]
		miR‐141‐3p/ZEB2 and E2F3	In vitro and clinical sample [41]
	SNHG16	let‐7b‐5p/CDC25B	In vitro, in vivo, and clinical sample [42]
		miR‐17‐5p/p62/mTOR and NF‐κB	In vitro, in vivo, and clinical sample [43]
	SNHG17	LRPPRC/c‐Myc	In vitro, in vivo, and clinical sample [44]
		P57	In vitro, and clinical sample [45]
Promoting EMT	SNHG1	EZH2/CDKN1A and CDKN2B, miR‐140‐5p/CDK4	In vitro, in vivo, and clinical sample [25]
		miR‐377‐3p	In vitro, in vivo, and clinical sample [46]
		miR‐376a/FOXK1//Snail	In vitro, in vivo, and clinical sample [47]
	GAS5	miR‐423–3p/SMARCA4	In vitro, in vivo, and clinical sample [18]
	SNHG3	miR‐128/CD151/PI3K/Akt	In vitro, and clinical sample [48]
		miR‐326/SMAD3/ZEB1	In vitro, in vivo, and clinical sample [49]
	SNHG4	miR‐211‐5p/CREB5	In vitro, in vivo, and clinical sample [50]
	SNHG5	miR‐26a‐5p/GSK3β/Wnt/β‐catenin	In vitro, in vivo, and clinical sample [31]
	SNHG6	miR‐101‐3p/ZEB1 and UPF1/TGF‐β/Smad	In vitro, in vivo, and clinical sample [34]
		SETD7/LZTFL1	In vitro and clinical sample [51]
	SNHG7	miR‐425/Wnt/β‐catenin	In vitro, in vivo, and clinical sample [52]
		miR‐122‐5p/FOXK2	In vitro, in vivo, and clinical sample [53]
	SNHG8	miR‐149‐5p/PPM1F	In vitro, in vivo, and clinical sample [54]
	SNHG12	miR‐516a‐5p/HEG1	In vitro and in vivo [38]
	DANCR	miR‐27a‐3p/LIMK1	In vitro, in vivo, and clinical sample [55]
	SNHG16	let‐7b‐5p/HMGA2	In vitro, in vivo, and clinical sample [42]
		miR‐4500/STAT3	In vitro and clinical sample [56]
		miR‐605‐3p/TRAF6/NF‐κB	In vitro, in vivo, and clinical sample [57]
	SNHG17	miR‐3180‐3p/RFX1	In vitro, in vivo, and clinical sample [58]
	SNHG20	EZH2/E‐cadherin	In vitro and clinical sample [59]
	MEG8 (SNHG23)	miR‐367‐3p/14–3‐3ζ/TGFβR1	In vitro and clinical sample [60]
Enhancing the stemness of HCC	SNHG3	METTL3/ITGA6	In vitro and clinical sample [61]
		miR‐502‐3p/YTHDF3/ITGA6 and HBXIP/METTL3/ITGA6	In vitro, in vivo, and clinical sample [62]
	SNHG5	UPF1/Wnt/β‐catenin	In vitro and in vivo [22]
	SNHG9	EZH2/PTEN	In vitro, in vivo, and clinical sample [63]
	SNHG12	Wnt/β‐catenin	In vitro [64]
	DANCR	miR‐214, miR‐320a, and miR‐199a/CTNNB1	In vitro, in vivo, and clinical sample [65]
Increasing HCC cell autophagy	SNHG11	miR‐184/AGO2	In vitro and clinical sample [66]
	DANCR	miR‐222‐3p/ATG7	In vitro and clinical sample [67]
	SNHG16	miR‐23b‐3p/EGR1	In vitro, in vivo, and clinical sample [68]
Suppressing autophagy	SNHG1	SLC3A2/Akt	In vitro and in vivo [69]
	SNHG29	Wnt/β‐catenin	In vitro [70]
Regulating ferroptosis	SNHG1/7	GPX4, NRF2, NCOA4, KEAP1, ACSBG1	In vitro [71]
		miR‐199a/FANCD2 and G6PD	In vitro, in vivo, and clinical sample [20]
Inhibiting ferroptosis	SNHG4	miR‐204‐5p/SNRPB2	In vitro [72]
Preventing the pyroptosis process	SNHG7	miR‐34a/SIRT1	In vitro and clinical sample [21]
Promoting aerobic glycolysis	SNHG1	miR‐326/PKM2	In vitro, in vivo, and clinical sample [73]
		SND1‐m6A‐SLC7A11	In vitro, in vivo, and clinical sample [24]
	SNHG6	BOP1	In vitro and clinical sample [74]
Regulating cholesterol metabolism	SNHG6	FAF2‐mTOR	In vitro, in vivo, and clinical sample [75]
Regulating fatty acid metabolism	SNHG1/7	ECHS1, MCEE, ACOT12, CPT1B, BDH2, CSBG1, FABP6, ACADVL, ACSM3, ACOX2, CPT2, ECI2, ECHS1, DECR, SLC27A6, MUT, SLC27A4, ACAD10, FASN, ACSL4, ACADSB, and GK2	In vitro [71]
Facilitating sorafenib resistance	SNHG1	SLC3A2/Akt	In vitro and in vivo [69]
		SND1‐m6A‐SLC7A11	In vitro, in vivo, and clinical sample [24]
	SNHG3	miR‐128/CD151/PI3K/Akt	In vitro, and clinical sample [48]
	SNHG4	miR‐204‐5p/SNRPB2	In vitro [72]
	DANCR	PSMD10‐IL‐6/STAT3	In vitro, in vivo, and clinical sample [76]
	SNHG16	miR‐140‐5p	In vitro, in vivo, and clinical sample [77]
		miR‐23b‐3p/EGR1	In vitro, in vivo, and clinical sample [68]
Reverses sorafenib resistance	GAS5	RBM38	In vitro and in vivo [78]
Improving cisplatin, and doxorubicin sensitivity	GAS5	miR‐21/PTEN	In vitro, in vivo, and clinical sample [16]
		miR‐222, VEGF, VEGFR	In vitro, and clinical sample [79]
Potentiating immunosuppressive microenvironment	SNHG6	ATF4, HnRNPA2B1, STAT6	In vitro, in vivo, and clinical sample [23]
	SNHG20	STAT6	In vitro, in vivo, and clinical sample [80]
Reprogramming the anti‐tumor immune microenvironment	GAS5	miR‐544/RUNX3	In vitro, in vivo, and clinical sample [81]
		PTEN	In vitro [82]
Promoting angiogenesis	SNHG14	PABPC1, PTEN	In vitro, in vivo, and clinical sample [83]
	SNHG16	miR‐4500/GALNT1/PI3K/Akt/mTOR	In vitro, in vivo, and clinical sample [84]
	SNHG22	EZH2/DNMT1/miR‐16‐5p	In vitro, in vivo, and clinical sample [85]

### 2.1 Impact of SNHGs on HCC Cell Proliferation by Regulating the Cell Cycle

**FIGURE 2:**
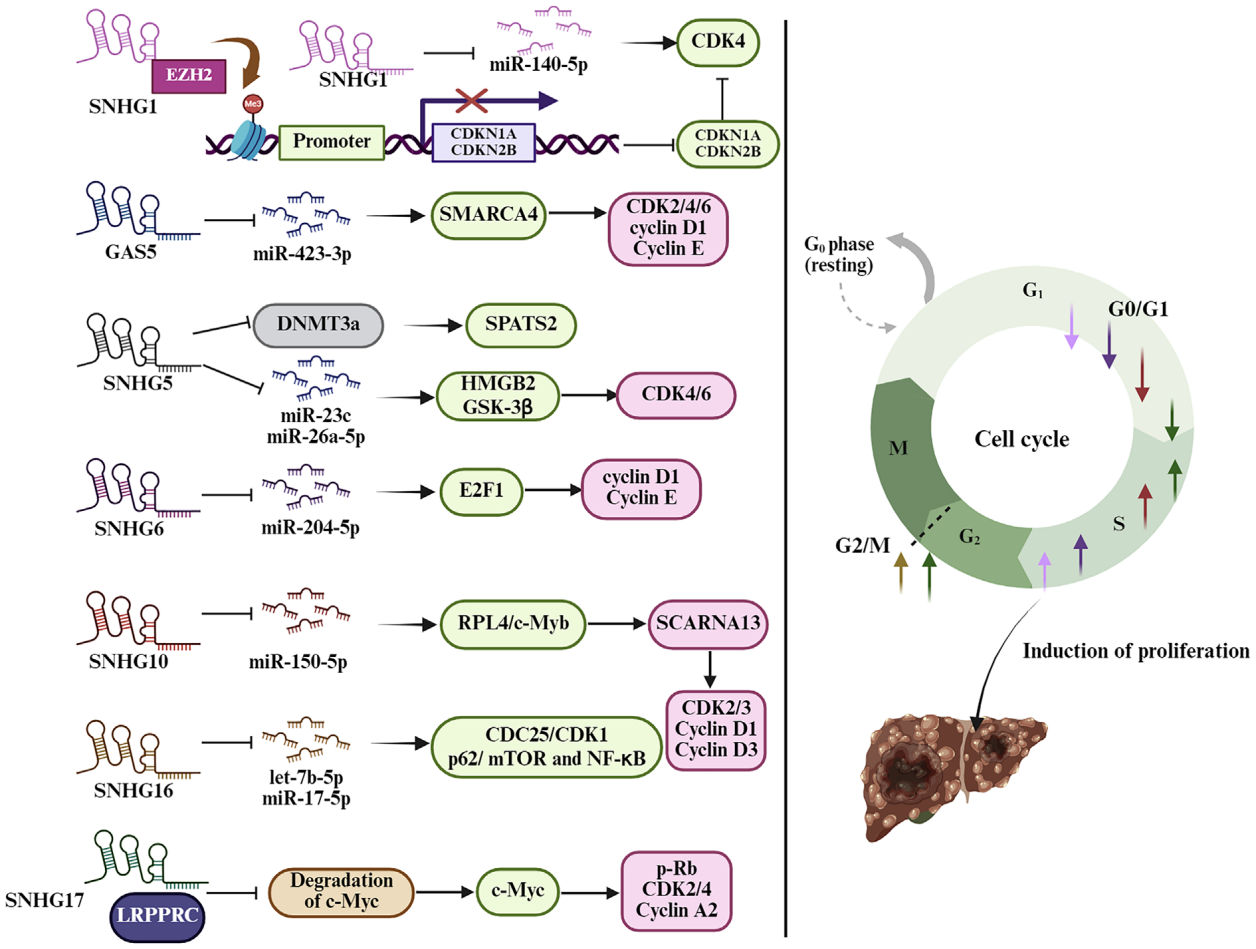
The roles of SNHGs in hepatocellular carcinoma cell proliferation by regulating the cell cycle. As depicted, SNHGs regulate proliferation‐related genes or pathways through epigenetic mechanisms, protein binding, or competing endogenous RNA networks, thus decreasing the proportion of liver cancer cells in the G0/G1 phase while increasing those in the S and G2/M phases, ultimately promoting the proliferation of liver cancer cells.

The regulation of the cell cycle plays a vital role in cancer biology, and SNHGs are significant contributors to this process in HCC. As shown in Figure 2 and Table 1, specific SNHGs, such as SNHG1, GAS5, SNHG5, SNHG6, SNHG10, SNHG16, and SNHG17, engage with various cell cycle regulators including CCND1, CDKN1A, CDKN2B, E2F1, E2F3, CDC25B, c‐Myc, cyclin A2/D1/D3 and CDK1/2/3/4/6. These interactions promote the S and/or G2/M transition, which ultimately leads to increased cancer cell proliferation [18, 25, 31, 32, 34, 42, 44]. Additionally, other SNHGs—including SNHG3, SNHG9, SNHG12, DANCR (Differentiation antagonizing non‐protein coding RNA, also known as SNHG13), and SNHG15—have been found to also regulate the cell cycle in HCC cells [28, 36, 38, 39, 40, 41]. However, the specific cell cycle‐related proteins involved remain unreported. The dysregulation of cell cycle control contributes to the uncontrolled growth characteristic of HCC, suggesting that SNHGs may serve as potential biomarkers for tumor aggressiveness and therapeutic targets for intervention.

### 2.2 Impact of SNHGs on HCC Cell Migration and Invasion Through EMT

**FIGURE 3:**
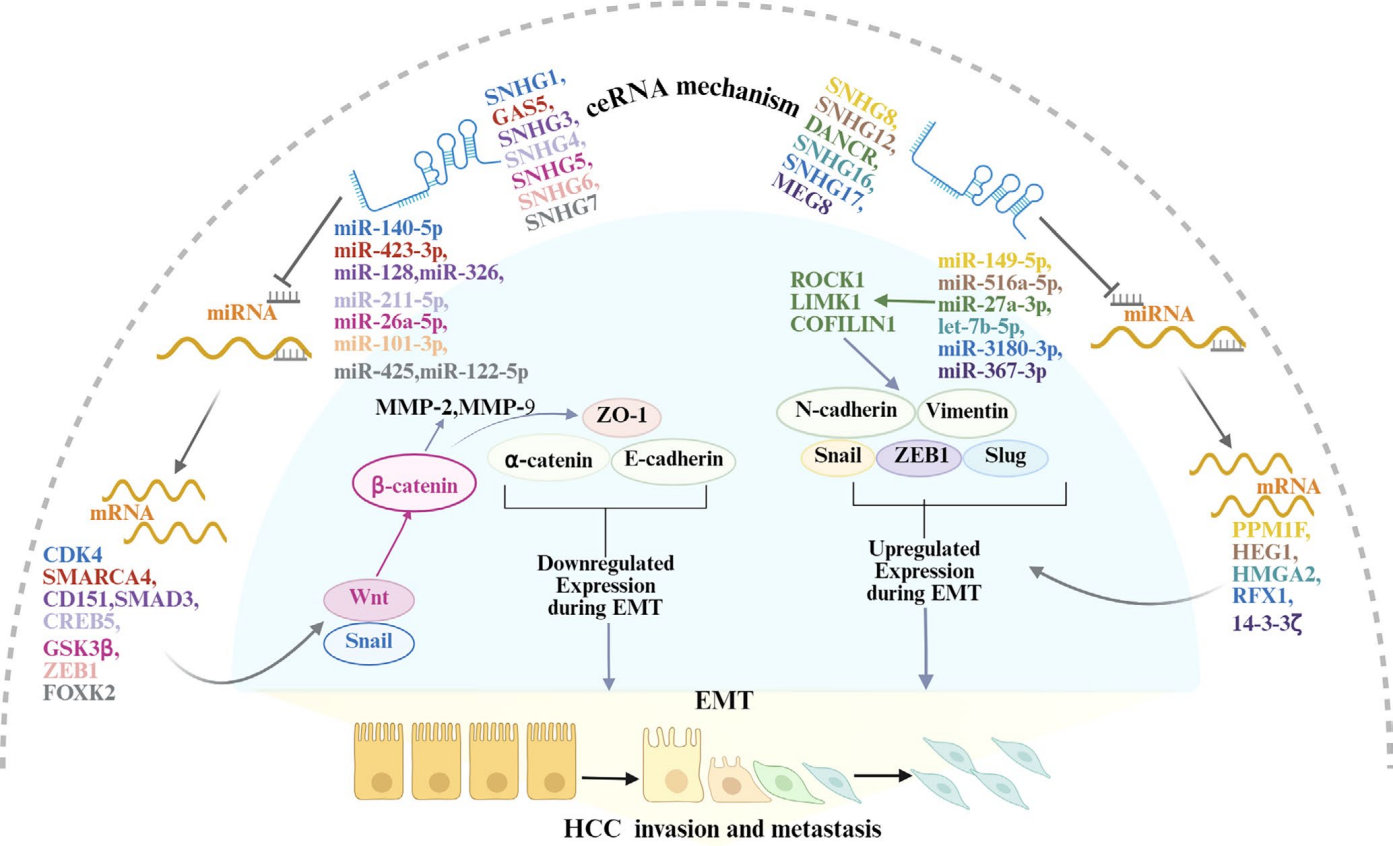
The roles of SNHGs in the invasion and metastasis of hepatocellular carcinoma cells through regulating epithelial‐mesenchymal transition (EMT). As depicted, the SNHG family members, especially SNHG1‐8, SNHG12, DANCR, SNHG16, SNHG17 and MEG8, have demonstrated the ability to enhance the invasion and metastasis of liver cancer cells. This is achieved through their interactions with a range of microRNAs, as well as various messenger RNAs.

Metastasis of HCC can impair multiple organ functions by invading extrahepatic organs (such as the lungs, bones, and brain), shorten patients' survival time, and seriously threaten their life and health. EMT (epithelial‐mesenchymal transition) plays a pivotal role in HCC metastasis by enhancing cell motility, invasion, and resistance to anoikis, ultimately facilitating the dissemination of tumor cells from the primary site [86]. Analysis of clinical samples revealed that the expression levels of SNHG1, SNHG3‐7, SNHG16, SNHG20, and MEG8 (Maternally expressed 8, also known as SNHG23) are associated with clinical indicators of liver cancer patients. These indicators include TNM stage, lymph node status, PVTT, and metastasis [31, 34, 47, 49, 50, 52, 53, 56, 57, 59, 60]. In vitro experiments showed that these SNHGs promote EMT in liver cancer cells by sponging miRNAs. This mechanism regulates the expression of downstream target genes, EMT‐related transcription factors, and proteins (as shown in Figure 3 and Table 1) [18, 25, 31, 34, 38, 42, 48, 49, 50, 52, 53, 54, 55, 58, 60]. Additionally, some in vivo experiments indicated that SNHG1, SNHG5, SNHG6, SNHG8, DANCR, SNHG16, and SNHG17 can promote lung metastasis of human liver cancer cells in immunodeficient mice [25, 31, 34, 42, 47, 54, 55, 57, 58]. Although GAS5 has been shown to promote EMT in liver cancer cells in vitro, further clinical data and in vivo experiments are needed to confirm their role in liver cancer metastasis [18]. These findings highlight the imperative to elucidate the mechanisms through which SNHGs promote tumor cell migration and invasion, as such insights are essential for developing effective therapeutic strategies.

### 2.3 Impact of SNHGs on HCC Cell Cancer Stemness

**FIGURE 4:**
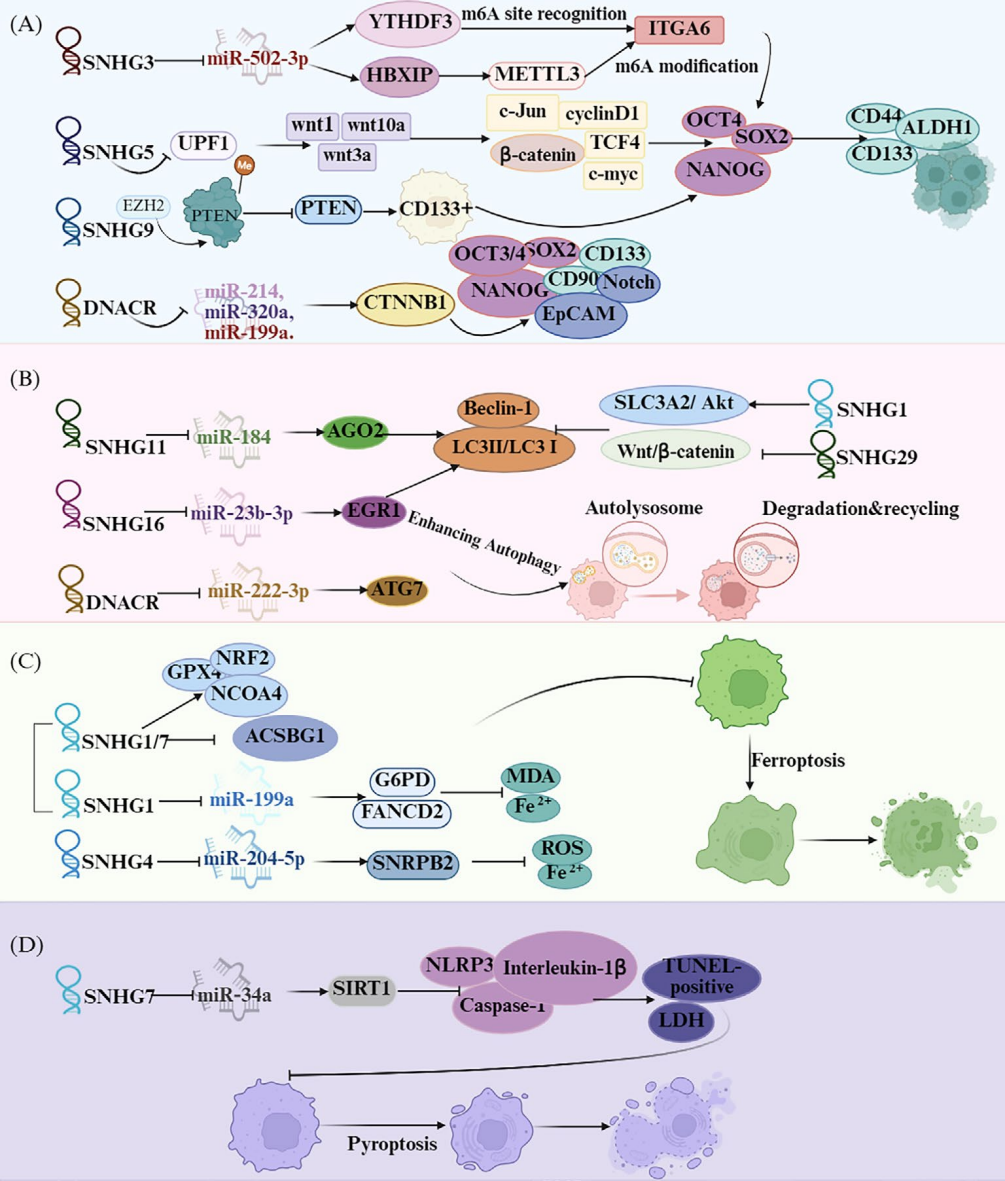
The roles of SNHGs in cancer stemness, autophagy, ferroptosis and pyroptosis in hepatocellular carcinoma (HCC). (A) LncRNA SNHGs promote liver cancer stemness. SNHG3, SNHG5, SNHG9 and DANCR can enhance the expression of ITGA6, Wnt/β‐catenin signaling pathway by inhibiting UPF1, PTEN and multiple miRNAs respectively, increasing the expression of various stemness‐related factors and markers, thereby enhancing the stemness of liver cancer cells. (B) LncRNA SNHGs regulate autophagy in HCC. SNHG11, DNACR and SNHG16 promote autophagy in liver cancer cells by sponging downstream microRNA to regulate the key autophagy‐related proteins such as Beclin‐1 and LC3. SNHG1 and SNHG29, in turn, inhibit autophagy in HCC cells by promoting the SLC3A2/Akt pathway and inhibiting the Wnt/β‐catenin pathway, respectively. (C) LncRNA SNHGs regulate ferroptosis in hepatocellular carcinoma. SNHG1 and SNHG7 dysregulate ferroptosis‐related proteins (GPX4, NRF2, NCOA4, ACSBG1, G6PD, and FANCD2), thereby modulating ferroptosis in liver cancer cells. SNHG4 inhibits the production of reactive oxygen species (ROS) and iron ions in HCC cells through the miR‐204‐5p/SNRPB2 axis. This inhibition suppresses ferroptosis. (D) SNHG7 suppresses miR‐34a expression, thereby modulating the expression of pyroptosis‐related genes (SIRT1, NLRP3, caspase‐1, and interleukin‐1β), ultimately inhibiting pyroptosis in liver cancer cells.

Cancer stemness is characterized by a specific group of cells within a tumor that exhibit stem cell‐like characteristics, such as the capacity for self‐renewal and the differentiation into multiple cell lineages [87]. In vitro colony formation and tumor sphere formation assays showed that SNHG3, SNHG5, SNHG9, and DNACR enhance the stem‐like properties of liver cancer stem cells. They also upregulate the expression of stem cell marker proteins (CD133, CD44, and ALDH1) and stemness‐associated genes (Oct4, Sox2, and Nanog) (see Table 1 and Figure 4A) [22, 61, 62, 63, 65]. Furthermore, in vivo experiments demonstrated that knocking down SNHG3, SNHG5, SNHG9, and DNACR inhibits the growth of liver cancer stem cells or reduces the expression of stem cell marker proteins in tumors [22, 61, 62, 63, 65]. Additionally, silencing SNHG12 decreases the expression of stem cell marker proteins (CD133, CD44, Nanog, LGR5, EpCAM) in liver cancer cells [64]. However, more evidence from tumor sphere formation assays and in vivo experiments is needed to clarify the specific role of SNHG12 in regulating the stemness of liver cancer cells. These stemness characteristics are associated with a more aggressive tumor phenotype and are often correlated with poor patient prognosis. Therefore, targeting these SNHGs may not only help in reducing tumor growth but also in diminishing the stem cell‐like properties of HCC cells, potentially leading to improved therapeutic outcomes (Table 2).

**TABLE 2:** Glossary of abbreviations.

Abbreviations	Full form
14–3‐3ζ	14–3‐3 Zeta Protein
ABCG2	ATP‐Binding Cassette Subfamily G Member 2
ACAD10	Acyl‐CoA Dehydrogenase Family Member 10
ACADSB	Acyl‐CoA Dehydrogenase, Short/branched Chain
ACADVL	Acyl‐CoA Dehydrogenase, Very Long Chain
ACSL4	Acyl‐CoA Synthetase Long Chain Family Member 4
ACSM3	Acyl‐CoA Synthetase Medium Chain Family Member 3
ACSBG1	Acyl‐CoA Synthetase Bubblegum Family Member 1
ACOX2	Acyl‐CoA Oxidase 2, Peroxisomal
AGO2	Argonaute 2
AFP	Apha‐fetoprotein
ASOs	Antisense oligonucleotides
ATF2/4	Activating Transcription Factor 2/4
ATG7	Autophagy Related 7
BDH2	3‐Hydroxybutyrate Dehydrogenase 2
BOP1	Block Of Proliferation 1
BUB1	Budding Uninhibited By Benzimidazoles 1
CCNA2/B1/D1	Cyclin A2/B1/D1
CDC25B	Cell division cycle 25B
CDKN1A/2B	Cyclin Dependent Kinase Inhibitor 1A/2B
CDK1/2/3/4/6	Cyclin Dependent Kinase 1/2/3/4/6
ceRNA	Competing endogenous RNA
CI	Confidence interval
CPT1B/2	Carnitine Palmitoyltransferase 1B, Muscle
CNNM1	Cyclin and CBS Domain Divalent Metal Cation Transport Mediator 1
CREB5	cAMP responsive element binding protein 5
CRISPR/Cas9	Clustered Regularly Interspaced Short Palindromic Repeats/CRISPR‐Associated Protein 9
CSBG1	Acyl‐CoA Synthetase Bubblegum Family Member 1, same as ACSBG1
CYP2C8	Cytochrome P450 family 2 subfamily C member 8
DECR	2,4‐Dienoyl‐CoA Reductase, Mitochondrial
DANCR	Differentiation antagonizing non‐protein coding RNA
DNMTs	DNA methyltransferases
DFS	Disease‐free survival
E2F1/3	E2F Transcription Factor 1/3
ECHS1	Enoyl‐CoA Hydratase, Short Chain 1, Mitochondrial
ECI2	Enoyl‐CoA Isomerase 2, Mitochondrial
EGR1	Early Growth Response 1
FANCD2	Fanconi Anemia Complementation Group D2
FASN	Fatty Acid Synthase
FABP6	Fatty Acid Binding Protein 6, Ileal
FGF19	Fibroblast Growth Factor 19
FAF2	FAS‐Associated Factor 2
FOXK1/2	Forkhead box K1/2
G6PD	Glucose‐6‐Phosphate Dehydrogenase
GAS5	Growth Arrest‐Specific 5
GALNT1	UDP‐N‐acetylgalactosamine transferase 1
GSTP1	Glutathione S‐Transferase P1
GSK3β	Glycogen Synthase Kinase 3β
GK2	Glycerol Kinase 2, Mitochondrial
GPX4	Glutathione Peroxidase 4
H3K27	Histone H3 Lysine 27
HBXIP	HBx‐Interacting Protein
HCC	Hepatocellular carcinoma
HDAC2	Histone Deacetylase 2
HEG1	Heart of Glass homolog 1
HIF1A	Hypoxia Inducible Factor 1 Alpha
HMGA2/B2	High Mobility Group A2/B2
HR	Hazard ratio
HNRNPL	Heterogeneous nuclear ribonucleoprotein L
HnRNPA2B1	Heterogeneous Nuclear Ribonucleoprotein A2B1
HOTAIR	HOX Transcript Antisense RNA
HUVECs	Human umbilical vein endothelial cells
IC_50_	Half‐maximal inhibitory concentration
IGF2BP2	IGF2 mRNA Binding Protein 2
ITGA6	Integrin subunit alpha 6
KIF11	Kinesin Family Member 11
KLF5/12	Kruppel‐like Factor 5/12
LIMK1	LIM Domain Kinase 1
LMNB2	Lamin B2
LMO4	LIM Only Protein 4
LncRNAs	Long non‐coding RNAs
LZTFL1	Leucine Zipper Transcription Factor Like 1
LRPPRC	Leucine‐Rich PPR Motif‐Containing Protein
MALAT1	Metastasis‐Associated Lung Adenocarcinoma Transcript 1
MAP3K13	Mitogen‐Activated Protein Kinase Kinase Kinase 13, same as MLLK3
MAT2A	Methionine Adenosyltransferase II, Alpha
MBD1	Methyl‐CpG binding domain protein 1
METTL3	Methyltransferase Like 3
MCEE	Methylmalonyl‐CoA Epimerase
miRNAs	microRNAs
MMP9	Matrix Metallopeptidase 9
MLLK3	Mixed Lineage Leukemia Kinase 3
MTDH	Metadherin
MRP1	Multidrug Resistance‐Associated Protein 1
MUT	Methylmalonyl‐CoA Mutase
NAFLD	Non‐alcoholic fatty liver disease
NEAT1	Nuclear Enriched Abundant Transcript 1
NEIL3	Nei Endonuclease VIII‐Like 3
NMD	Nonsense‐mediated decay
NCAPG	Non‐SMC Condensin I Complex Subunit G
NLRP3	NLR family pyrin domain containing 3
NCOA4	Nuclear Receptor Coactivator 4
NRF2	Nuclear Factor, Erythroid 2 Like 2
OS	Overall survival
PABPC1	Polyadenylate‐Binding Protein 1
PDCD4	Programmed Cell Death 4
PDHA1	Pyruvate Dehydrogenase E1 Alpha 1
P‐gp	P‐glycoprotein, ATP‐Binding Cassette Subfamily B Member 1
PKM2	Pyruvate Kinase isozyme type M2
PPM1F	Protein Phosphatase, Mg^2+^/Mn^2+^ Dependent 1F
PTEN	Phosphatase And Tensin Homolog
PTBP1	Polypyrimidine tract‐binding protein 1
PSMD10	Proteasome 26S Subunit, Non‐ATPase 10
PVTT	Portal vein tumor thrombosis
RECK	Reversion‐inducing‐cysteine‐rich protein with kazal motifs
RFX1	Regulatory Factor X 1
RFS	Recurrence‐free survival
RIP	RNA immunoprecipitation
RNF38	Ring Finger Protein 38
ROCK1	Rho associated coiled‐coil containing protein kinase 1
ROS	Reactive oxygen species
RPL4/S3	Ribosomal Protein L4/S3
RSL‐3	Ras Selective Lethal 3
RUNX3	Runt‐related transcription factor 3
RT‐PCR	Reverse transcription‐polymerase chain reaction
SCARNA13	Small Cajal Body‐Specific RNA 13
SETD7	SET Domain Containing 7, Histone Lysine Methyltransferase
SERPINH1	Serpin Family H Member 1
SIRT1/5	Sirtuin 1/5
SLC27A4/6	Solute Carrier Family 27 Member 4/6
SLC3A2	Solute Carrier Family 3 Member 2
SLC7A11	Solute carrier family 7 member 11
SND1	Staphylococcal Nuclease Domain‐Containing Protein 1
siRNAs	Small interfering RNAs
snoRNAs	Small nucleolar RNAs
SNRPB2	Small nuclear ribonucleoprotein polypeptide B2
SOCS1	Suppressor of Cytokine Signaling 1
SMAD3	SMAD family member 3
SMARCA4	SWI/SNF Related, Matrix Associated, Actin Dependent Regulator Of Chromatin, Subfamily A, Member 4
SOX9	Recombinant Sex Determining Region Y Box Protein 9
SPATS2	Sperm‐Associated Antigen 2
SNHGs	Small nucleolar host genes
SSR2	Signal Sequence Receptor Subunit 2
STAT3/6	Signal Transducer And Activator Of Transcription 3/6
TGFβR1	Transforming Growth Factor Beta Receptor 1
TOP2A	Topoisomerase II Alpha
TRAF6	Tumor Necrosis Factor Receptor‐Associated Factor 6
UPF1	Up‐frameshift protein 1
VEGF	Vascular endothelial growth factor
VEGFR	Vascular Endothelial Growth Factor Receptor
ZEB1/2	Zinc Finger E‐Box Binding Homeobox 1/2

### 2.4 Impact of SNHGs on HCC Cell Autophagy

Autophagy is a cellular degradation process that has two opposing roles in cancer: it acts as a tumor suppressor in the early stages but promotes survival in established tumors [88]. In HCC, SNHGs have been identified as key regulators of autophagy, which affects both tumor progression and the response to therapy (Figure 4B) [66, 67, 68, 69, 70]. For example, the SNHG11/miR‐184/AGO2 and DANCR/miR‐222‐3p/ATG7 regulatory loop has been shown to enhance autophagic flux in liver cancer cells, which helps these cells survive under stressful conditions [66, 67]. Additionally, research by Jing Z et al. demonstrated that overexpressing SNHG16 increases both the viability of Hep3B/So (sorafenib‐resistant) cells and their autophagy levels. This effect is mediated through the regulation of crucial autophagy‐associated proteins, including Beclin‐1 and LC3, both of which are vital for the formation and maturation of autophagosomes [68]. The aforementioned SNHGs promote autophagy; they also enhance cell proliferation and tumor growth. These findings suggest that the autophagy induced by these SNHGs is likely cytoprotective and exerts an oncogenic role.

However, in sorafenib‐resistant HCC cells, cytoprotective autophagy can switch to cytotoxic autophagy under certain conditions [89]. Strengthening autophagy in these drug‐resistant cells therefore produces an anti‐tumor effect. Li W et al. observed high SNHG1 expression in sorafenib‐resistant cells; inhibiting SNHG1 boosts autophagy and apoptosis in these cells, while also suppressing in vivo tumor growth [69]. This indicates that SNHG1 may counteract cytotoxic autophagy, thereby contributing to sorafenib resistance in HCC cells. Additionally, studies have demonstrated that resveratrol inhibits HCC cell proliferation and migration while upregulating SNHG29 expression [70]. Although SNHG29 can inhibit autophagy in HCC cells, its role in mediating resveratrol's anti‐HCC effects remains unclear and warrants further investigation.

The elevated levels of SNHGs in HCC are associated with altered autophagic activity, potentially contributing to tumorigenesis and progression of HCC and the resistance of liver cancer cells against sorafenib and other chemotherapy. Therefore, targeting the SNHG‐autophagy pathway may offer a promising strategy to enhance the sensitivity of HCC cells to various therapies.

### 2.5 Impact of SNHGs on HCC Cell Ferroptosis

Ferroptosis is a type of regulated cell death driven by iron‐dependent lipid peroxidation, and it has emerged as a promising therapeutic target in cancer research [90]. Recent investigations have highlighted the role of SNHGs in modulating ferroptosis in HCC cells (Figure 4C and Table 1) [20, 71, 72]. Specifically, the silencing of SNHG1 or SNHG7 in HepG2 and Huh7 cell lines has been shown to reduce intracellular lipid droplets. It also downregulates the expression of ferroptosis‐inhibiting proteins (GPX4 and NRF2) and the ferroptosis‐promoting protein NCOA4, while upregulating the expression of the ferroptosis‐promoting protein ACSBG1 [71]. These findings suggest that SNHG1 and SNHG7 may play a regulatory role in ferroptosis processes within HCC cells; however, validation using ferroptosis markers such as intracellular iron ion concentration and lipid peroxidation levels in liver cancer cells remains lacking. The role and mechanism of SNHG1 and SNHG7 in ferroptosis thus require further investigation. Notably, Zhou L et al. demonstrated that knocking down SNHG1 increases intracellular lipid peroxidation and Fe^2+^ concentration in HCC cells, promoting erastin‐induced ferroptosis [20]. This indicates that SNHG1 can inhibit ferroptosis to protect HCC cells, though additional confirmation (e.g., in vivo experiments) is needed to clarify SNHG1's effect on ferroptosis in these cells. Another study discovered that SNHG4 upregulates SNRPB2 expression in liver cancer cells via a competing endogenous RNA (ceRNA) mechanism. This upregulation reverses the increased lipid peroxidation, elevated Fe^2+^ ion concentration, and tumor‐suppressive effects induced by the ferroptosis promoter RSL‐3 following SNRPB2 knockdown, thereby promoting sorafenib resistance in liver cancer cells [72]. The interaction between SNHGs and ferroptosis pathways unveils a novel strategy that liver cancer cells may employ to avoid cell death, thereby facilitating tumor growth and resistance to treatment. A thorough understanding of the role of SNHGs in modulating ferroptosis may lead to innovative therapeutic strategies aimed at triggering ferroptosis in HCC cells, potentially overcoming resistance to conventional treatments.

### 2.6 Impact of SNHGs on HCC Cell Pyroptosis

Pyroptosis, a distinct form of programmed cell death closely associated with inflammatory responses, plays a dual role in cancer by inhibiting tumor growth while also promoting tumor progression via inflammation [91]. SNHGs can regulate pyroptosis‐related gene expression via the ceRNA mechanism, thereby influencing the proliferation, invasion, and metastasis of liver cancer cells (Figure 4D) [21]. For example, SNHG7 may play an indirect role in modulating the function of key pyroptosis‐related molecules, including sirtuin 1 (SIRT1), NLR family pyrin domain containing 3 (NLRP3), caspase‐1, and interleukin‐1β, by interacting with and suppressing miR‐34a, thereby preventing the pyroptosis process in HCC cells [21]. Further research is still needed to clarify the role of pyroptosis inhibited by SNHG7 in the tumorigenesis and progression of HCC. Exploring the regulatory mechanisms of SNHGs on pyroptosis in HCC is essential, as it not only sheds light on the molecular mechanisms underlying HCC but also opens up potential new avenues for targeted therapies in liver cancer treatment.

### 2.7 Impact of SNHGs on Glucose, Fatty Acid and Cholesterol Metabolism in HCC Cells

**FIGURE 5:**
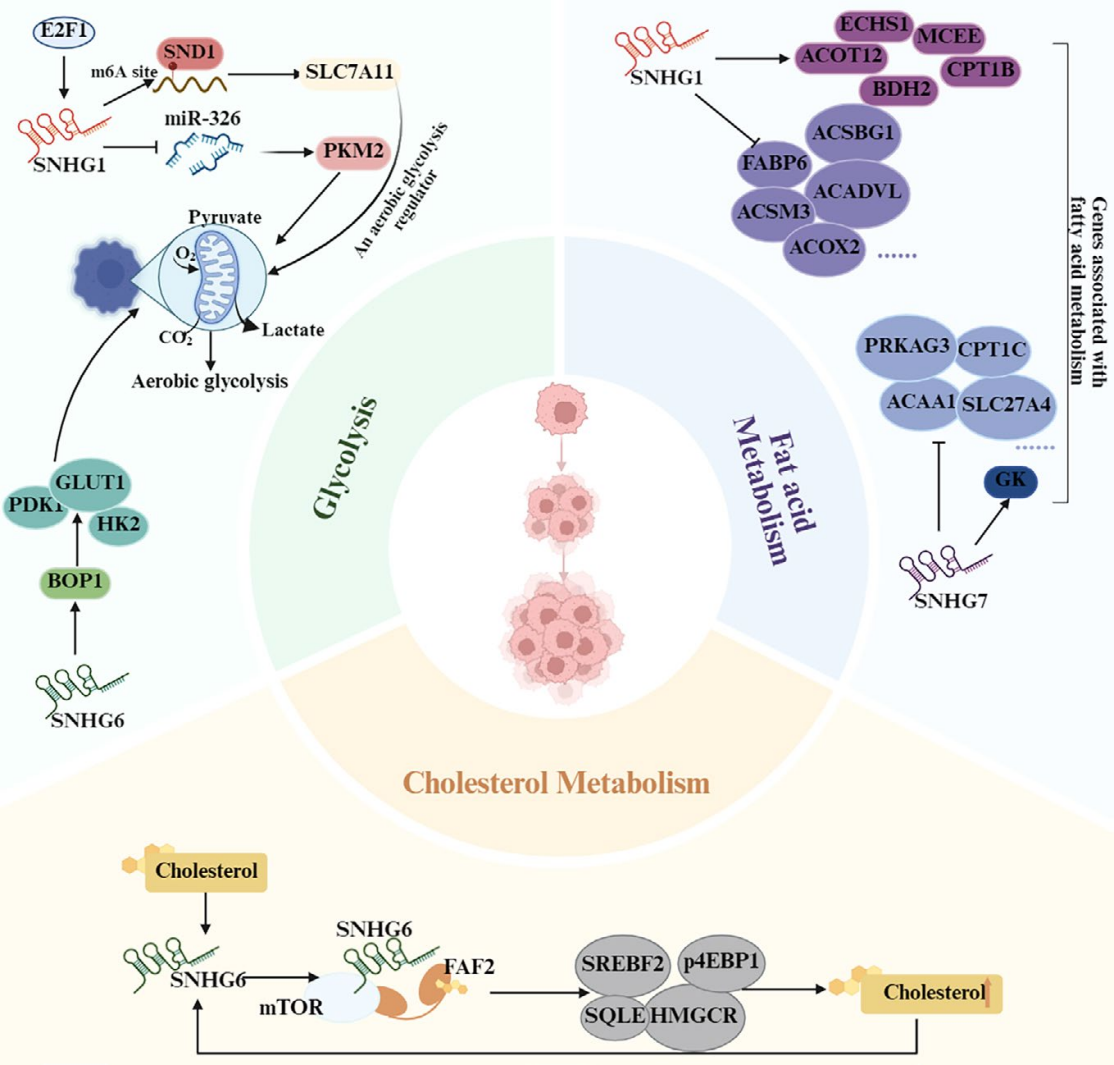
The roles of SNHGs in glucose, fatty acid, and cholesterol metabolism in hepatocellular carcinoma. SNHG1 and SNHG6 upregulate glycolytic enzymes to enhance glycolysis, while SNHG1 and SNHG7 modulate multiple genes in fatty acid metabolism—potentially regulating this pathway. In contrast, SNHG6 forms a complex with FAF2 and mTOR to promote phosphorylation of cholesterol biosynthesis‐related proteins, thereby stimulating cholesterol synthesis.

Altered glucose metabolism, commonly referred to as the Warburg effect, serves as a fundamental hallmark of cancer. In this process, cancer cells preferentially rely on glycolysis for energy generation, even in the presence of oxygen [92]. SNHGs play a role in regulating glucose metabolism in liver cancer cells (as illustrated in Figure 5) [24, 73, 74]. Studies have demonstrated that overexpression of SNHG1 increased glucose uptake and lactate production, and decreased the ratio of oxygen consumption rate (OCR)/extracellular acidification rate (ECAR) in HCC cells. An inhibitor of the glycolysis pathway, 2‐DG treatment weakened the proproliferative effect and sorafenib resistance in SNHG1 overexpression HCC cells. These findings suggested that SNHG1 contributes to tumor promotion and sorafenib resistance in HCC, at least in part, by activating the glycolytic pathway [24, 73]. Furthermore, research conducted by Chen K et al. demonstrated that SNHG6 enhances cell proliferation, inhibits apoptosis, increases glucose uptake and lactate production, reduces the oxygen consumption rate, and raises the extracellular acidification rate in HCC cell lines [74]. This metabolic reprogramming enables HCC cells to satisfy their increased energy demands and supports rapid proliferation. The involvement of SNHGs in regulating glucose metabolism underscores their potential as therapeutic targets. Targeting and inhibiting their expression may disrupt the metabolic adaptations that promote HCC progression.

Fatty acids are essential for synthesizing cell membranes, and dysregulated fatty acid metabolism is closely associated with the onset and development of HCC [93]. Research conducted by Chen E et al. has shown that SNHG1/7 can affect the expression of various genes associated with fatty acid metabolism, including ECHS1, MCEE, ACOT12, CPT1B, BDH2, CSBG1, FABP6, ACADVL, ACSM3, ACOX2, CPT2, ECI2, ECHS1, DECR, SLC27A6, MUT, SLC27A4, ACAD10, FASN, ACSL4, ACADSB, and GK2 (Figure 5) [71]. This finding implies that SNHG1/7 may influence the initiation and progression of HCC by regulating fatty acid metabolism, but the specific mechanisms still require further exploration.

Dysregulated cholesterol metabolism is a hallmark of HCC, with increased cholesterol synthesis, altered storage, and impaired efflux contributing to tumor progression [94]. Liu F discovered that cholesterol stimulates the expression of SNHG6 which in turn promotes the interaction between SNHG6 and the endoplasmic reticulum‐anchored protein FAF2, as well as with mTOR, resulting in the formation of a ternary complex (SNHG6‐FAF2‐mTOR). This complex facilitates the recruitment and activation of mTORC1 on the lysosomal surface, thereby driving cholesterol synthesis, cell proliferation, and tumor progression. As a result, the interplay between cholesterol and SNHG6 establishes a positive feedback mechanism. Knockdown of SNHG6 markedly inhibits the phosphorylation of mTORC1 downstream target proteins (e.g., SREBF2, 4EBP1), reducing cholesterol synthesis and cell viability. Further studies demonstrated that overexpression of SNHG6 accelerates the malignant transformation from non‐alcoholic fatty liver disease (NAFLD) to HCC in a mouse model induced by a high‐cholesterol diet [75]. In conclusion, SNHG6 plays a central role in cholesterol‐driven hepatocarcinogenesis by regulating key nodes of cholesterol metabolism (synthesis, distribution, and signaling), providing a novel strategy for targeted therapy.

### 2.8 Regulation of Drug Sensitivity by SNHGs in HCC

**FIGURE 6:**
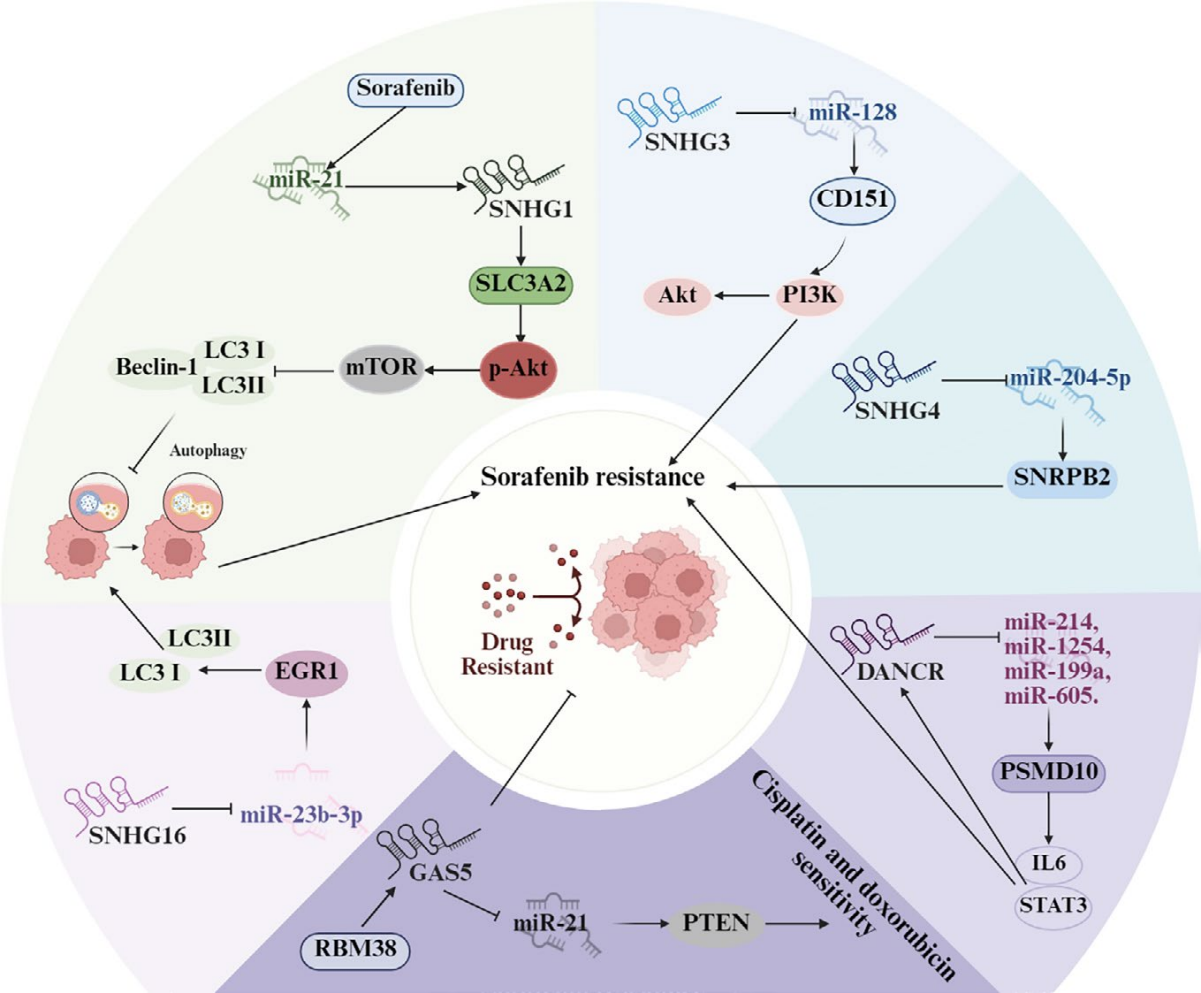
The roles of SNHGs in sorafenib resistance in hepatocellular carcinoma (HCC). SNHG1 and SNHG16 modulate autophagy in HCC cells through the Akt/mTOR pathway and miR‐23b‐3p/EGR1 axis, respectively, which contributes to their resistance to sorafenib. SNHG3, SNHG4, and DANCR also enhance sorafenib resistance in HCC cells by competitively binding ceRNA networks to activate the PI3K/Akt and IL6/STAT3 signaling pathways, respectively. However, GAS5 enhances the sensitivity of HCC cells to sorafenib, doxorubicin, and cisplatin.

Sorafenib is recognized as a primary pharmacological agent utilized in the systemic management of HCC. However, the development of resistance to sorafenib significantly hampers its therapeutic efficacy in treating HCC [95]. As mentioned above, particular SNHGs such as SNHG1, SNHG4, and SNHG16 can contribute to the resistance of HCC cells to sorafenib by regulating autophagy, ferroptosis, and glycolysis [24, 68, 69, 72]. Furthermore, a study conducted by Zhang PF and colleagues revealed that patients exhibiting elevated levels of SNHG3 had significantly diminished overall survival rates post‐sorafenib treatment when compared to those with lower SNHG3 expression. They also identified that the half‐maximal inhibitory concentration (IC_50_) for sorafenib in SNHG3‐overexpressing liver cancer cells (PLC/PRF/5) was considerably higher than that in control cells [48]. Additionally, Liu Y et al. demonstrated that DANCR was markedly upregulated in sorafenib‐resistant HCC cells. Knocking down DANCR significantly enhances the sensitivity of these cells to sorafenib treatment, both in vitro and in vivo [76]. These studies suggest that SNHG3 and DANCR can also promote sorafenib resistance in HCC cells via the ceRNA mechanism (see Figure 6). However, whether they also regulate processes such as autophagy, ferroptosis, or glycolysis to promote resistance remains to be further investigated.

However, studies have shown that knocking down GAS5 in sorafenib‐resistant cell lines with RBM38 overexpression increases sorafenib's IC_50_ value, inhibits cell apoptosis, elevates the proportion of cells in the S phase, and promotes both the in vivo proliferation of resistant cells and the expression of drug resistance‐related proteins (e.g., P‐gp, MRP1, ABCG2) [78]. Other studies have also indicated that GAS5 can sponge miRNAs to overcome resistance to doxorubicin and cisplatin [16, 79]. Collectively, these findings suggest that GAS5 exerts an inhibitory effect on HCC tumorigenesis and progression, while also enhancing the sensitivity of HCC cells to drugs such as sorafenib, doxorubicin, and cisplatin. This contradicts the findings of Kim SY's study [18], which may be attributed to the fact that the GAS5 molecules examined in these studies are distinct transcripts.

The above studies suggest that several SNHG family members contribute substantially to HCC cell resistance to therapeutic drugs. Elucidating the mechanisms of SNHGs' influence on drug resistance is essential for improving therapeutic outcomes and developing effective strategies to counteract resistance in HCC.

### 2.9 Impact of SNHGs on Tumor Microenvironment and Angiogenesis in HCC

**FIGURE 7:**
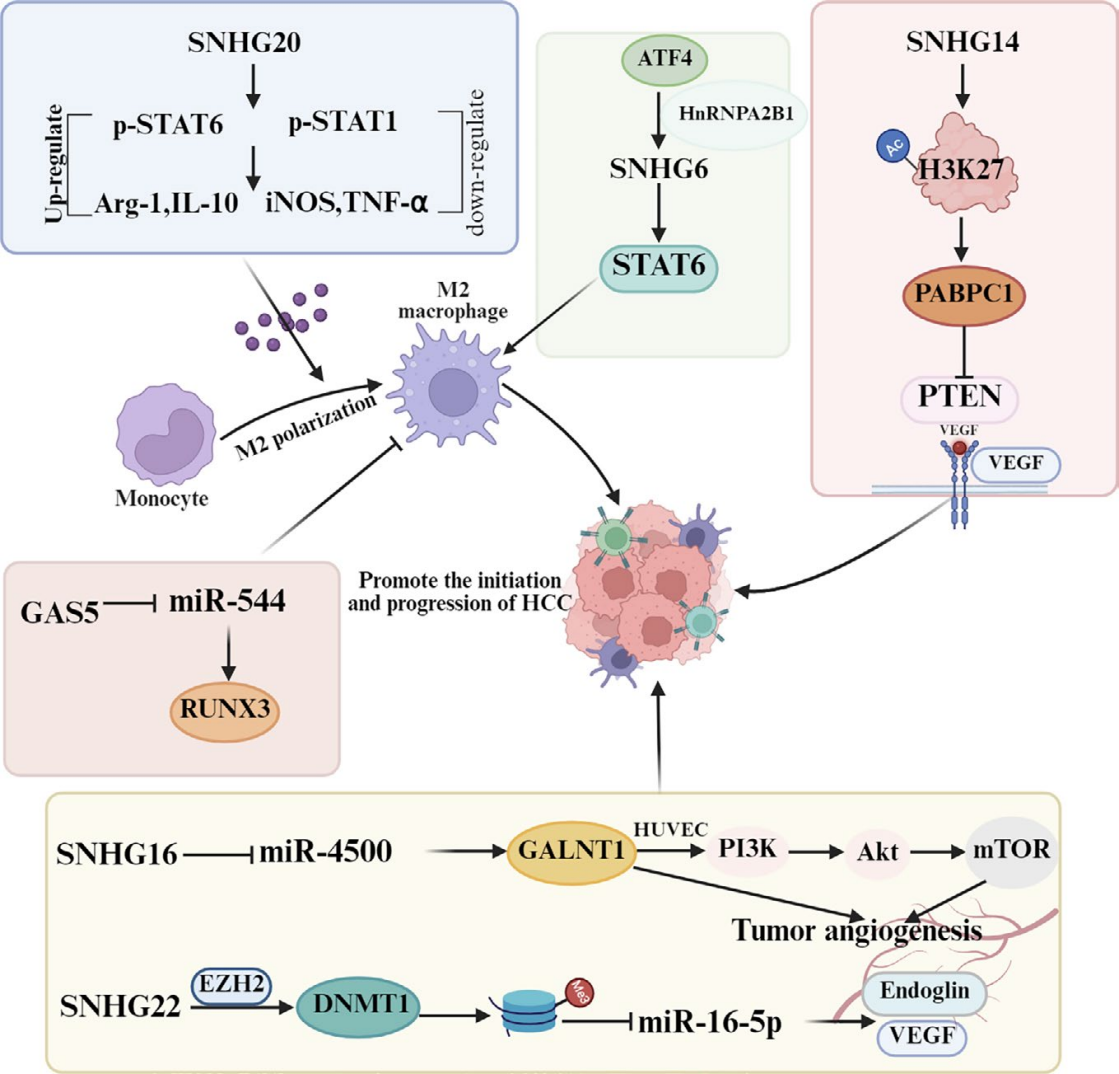
The roles of SNHGs in tumor microenvironment and angiogenesis in hepatocellular carcinoma (HCC). SNHG20 and SNHG6 facilitate HCC tumorigenesis and progression by promoting M2 polarization through upregulating STAT6 phosphorylation. GAS5, in turn, inhibits M2 polarization via the miR‐544/RUNX3 axis, thereby enhancing anti‐tumor immunity against HCC. Meanwhile, SNHG14, SNHG16, and SNHG22 promote angiogenesis in liver cancer by enhancing VEGF expression via regulating histone acetylation, miR‐4500/GALNT1/PI3K/Akt/mTOR pathway and miRNA promoter methylation, respectively.

The tumor microenvironment is critical in the progression of HCC. For instance, the shift in macrophage polarization from the M1 to the M2 phenotype promotes the onset and progression of HCC [96]. Notably, as shown in Figure 7, SNHGs can influence HCC progression through their regulatory effects on the tumor microenvironment. Studies have shown that endoplasmic reticulum stress induced by ATF4 (Activating Transcription Factor 4) overexpression drives the release of SNHG6 into exosomes via HnRNPA2B1 (Heterogeneous Nuclear Ribonucleoprotein A2B1). Once released, SNHG6 enhances M2 polarization through STAT6 and impairs the cytotoxic activity of CD8^+^ T cells. Knocking down SNHG6 suppresses macrophage M2 polarization, endoplasmic reticulum stress, and tumor growth—both in vitro and in vivo—and also boosts the anti‐tumor efficacy of anti‐PD‐1 therapy [23]. The same line of research also indicated that SNHG20 promotes M2 polarization via STAT6, thereby accelerating the progression of NAFLD to HCC [80].

In contrast, other studies found that GAS5 increases the cytotoxicity of NK cells against HCC cells and inhibits macrophage M2 polarization—via the miR‐544/RUNX3 axis and PTEN, respectively—thus enhancing anti‐tumor immune function [81, 82].

Collectively, these findings demonstrate that SNHGs can both foster an immunosuppressive microenvironment and reshape the anti‐tumor immune microenvironment in HCC. Gaining insights into the interactions between SNHGs and the tumor microenvironment is essential for developing effective therapeutic strategies that target both the tumor itself and its surrounding supportive environment.

Additionally, SNHGs can influence the composition of the extracellular matrix and stimulate angiogenesis (Figure 7). For example, SNHG14 has been shown to markedly elevate the levels of polyadenylate‐binding protein 1 (PABPC1) via lysine 27 on histone H3 (H3K27) acetylation, subsequently enhancing both cell proliferation and angiogenesis through the PTEN signaling pathway, as evidenced in both in vitro and in vivo studies [83]. Another study has shown that exosome‐derived SNHG16 promotes angiogenesis in vascular endothelial cells in vitro via the miR‐4500/GALNT1/PI3K/Akt/mTOR pathway. It also enhances both tumor growth and angiogenesis of HCC cells in vivo [84]. On the other hand, the silencing of SNHG22 has been found to greatly reduce angiogenesis in human umbilical vein endothelial cells (HUVECs) by lowering the expression of vascular endothelial growth factor (VEGF) along with other angiogenesis‐promoting factors. Furthermore, the absence of SNHG22 has been correlated with decreased tumor growth and angiogenesis in vivo. This downregulation is thought to be mediated by the regulation of miR‐16‐5p [85]. Therefore, targeting SNHGs could be a promising strategy to disrupt the angiogenic processes that fuel HCC progression.

## 3 Possible Molecular Mechanisms of SNHGs Affecting HCC Development and Progression

The molecular mechanisms through which SNHGs influence HCC development mainly include four aspects: the modulation of gene expression via the ceRNA network, the involvement of snoRNAs, the processes of epigenetic regulation, and various additional mechanisms

### 3.1 Role of SNHGs in the CeRNA Network

**FIGURE 8:**
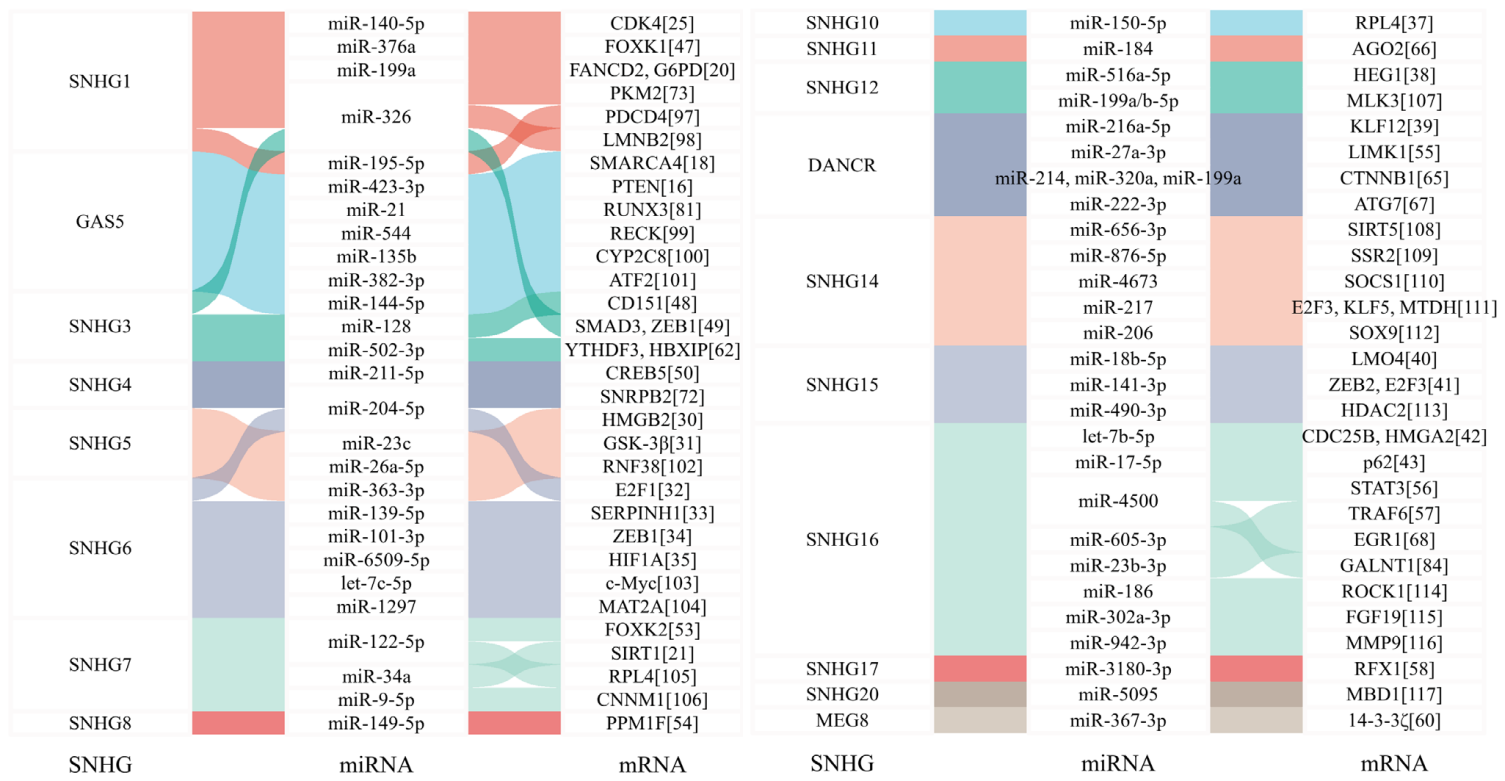
The competing endogenous RNA network associated with SNHGs by Sankey diagram. As shown, SNHGs compete with mRNAs for binding to the same miRNAs. This blocks the miRNAs from inhibiting their target mRNAs, in turn indirectly regulating downstream gene expression and influencing cellular processes.

The ceRNA network represents a regulatory mechanism where different RNA molecules compete for shared miRNA, thereby influencing gene expression. The mechanism by which SNHGs regulate the tumorigenesis of liver cancer has been most extensively studied in terms of SNHGs acting through the ceRNA network. SNHGs, ranging from SNHG1 to SNHG23, serve as pivotal components of these networks, as they can bind to miRNAs and prevent them from suppressing their target mRNAs. This interaction can lead to an upregulation of oncogenes or tumor suppressor genes in HCC. We summarize the SNHG/miRNA/mRNA networks that have been verified by dual‐luciferase reporter gene assays, RNA immunoprecipitation (RIP) assays, and/or reverse transcription‐polymerase chain reaction (RT‐PCR) through Sankey diagram in Figure 8 [16, 18, 20, 21, 25, 30, 31, 32, 33, 34, 35, 37, 38, 39, 40, 41, 42, 43, 47, 48, 49, 50, 53, 54, 55, 56, 57, 58, 60, 62, 65, 66, 67, 68, 72, 73, 81, 84, 97, 98, 99, 100, 101, 102, 103, 104, 105, 106, 107, 108, 109, 110, 111, 112, 113, 114, 115, 116, 117]. The dysregulation of SNHGs within the ceRNA landscape highlights their role in the pathogenesis of HCC. Moreover, it underscores their potential as biomarkers for prognosis and as therapeutic targets in HCC management.

### 3.2 Impact of SNHGs on Small Nucleolar RNA

SNHGs are not only involved in ceRNA interactions but also play a crucial role in the biogenesis and function of snoRNAs. These snoRNAs are vital for the modification of ribosomal RNA and various other RNA species, thereby affecting protein synthesis and overall cellular functions [14]. The dysregulation of SNHGs can lead to altered snoRNA expression, which may contribute to the malignant phenotype observed in HCC [118]. For instance, SCARNA13, a specific snoRNA, is produced from the intronic regions of the primary RNA transcript of the SNHG10 gene, while the corresponding exons are incorporated into the SNHG10 transcript. The silencing of SCARNA13 notably rescued the effects of SNHG10 overexpression on cellular proliferation, invasion, and migratory capabilities [37]. These results suggest that SCARNA13 mediates the tumor‐promoting function of SNHG10. The interaction between SNHGs and snoRNAs indicates that these molecules may work synergistically to promote tumorigenesis, underscoring their potential as therapeutic targets in HCC treatments.

### 3.3 Mechanisms of Epigenetic Regulation by SNHGs

SNHGs also play a significant role in epigenetic regulation, affecting gene expression through various mechanisms, such as histone modification and chromatin remodeling. Previous studies have shown that SNHG1, SNHG9, and SNHG20 can recruit enhancer of Zeste homolog 2 (EZH2) to the promoter regions of CDKN1A/CDKN2B, PTEN, and E‐cadherin, respectively. This recruitment drives the methylation of H3K27, which in turn reduces the expression levels of these genes and ultimately facilitates the proliferation, invasion, migration, and stemness maintenance of HCC cells [25, 59, 63].

Another study revealed that SNHG14 enhances histone acetylation of PABPC1 to upregulate PABPC1 expression; this, in turn, promotes the proliferation, migration, and angiogenesis of HCC cells. However, the specific mechanism by which SNHG14 promotes PABPC1 histone acetylation remains unclear [83].

Additionally, studies have confirmed that SNHG family members regulate gene expression by recruiting DNA methyltransferases (DNMTs), thereby contributing to HCC initiation and progression. For instance, in HCC cells, SNHG1 and SNHG9 can recruit DNMT1, DNMT3A, and DNMT3B to the promoter regions of p53 and glutathione S‐transferase P1 (GSTP1), respectively. This recruitment induces methylation of these promoter regions, leading to suppressed expression of p53 and GSTP1 [27, 36]. In contrast, SNHG5 inhibits DNMT3a activity, which reduces methylation of the sperm‐associated antigen 2 (SPATS2) promoter region. This reduction elevates SPATS2 expression in HCC cells and ultimately promotes their proliferation and migration [29]. Furthermore, a study found that in HCC, SNHG22 recruits DNMT1 to the promoter region of miR‐16‐5p via EZH2. This recruitment induces methylation of the miR‐16‐5p promoter, thereby inhibiting miR‐16‐5p expression [85].

Beyond regulating DNA and miRNA methylation, SNHG family members can also modulate gene expression through N6‐methyladenosine (m^6^A) RNA modification. For example, in HCC, SNHG1 upregulates the expression of the m^6^A RNA reader staphylococcal nuclease domain‐containing protein 1 (SND1), which further increases the mRNA expression level of solute carrier family 7 member 11 (SLC7A11). Nevertheless, the specific mechanism by which SNHG1 upregulates SND1 expression, as well as how SND1 modulates m^6^A modification of SLC7A11 mRNA, still requires further investigation [24].

The intricate interactions between SNHGs and epigenetic modifications highlight their potential as therapeutic targets, as modulating these interactions could restore normal gene expression patterns in HCC.

### 3.4 Impact of SNHGs on mRNA Stability

Studies show that SNHG family members regulate HCC initiation and progression by modulating RNA stability. Wang H et al. found that SNHG6 acts as a “decay factor” by competitively binding to heterogeneous nuclear ribonucleoprotein L (HNRNPL), thereby blocking HNRNPL's ability to stabilize SETD7 mRNA. Concurrently, SNHG6 functions as a “guide” to help polypyrimidine tract‐binding protein 1 (PTBP1) bind LZTFL1 mRNA and drive its degradation. The reduction in SETD7 and LZTFL1 expression further activates tumor‐associated EMT and genes related to migration and invasion, ultimately promoting the malignant phenotype of HCC cells [51].

Another study by Chang L et al. demonstrated that SNHG6 directly binds the RNA‐binding protein UPF1 (Up‐frameshift protein 1), which enhances the nonsense‐mediated decay (NMD) of Smad7 mRNA. The enhanced decay of Smad7 mRNA suppresses its expression, which in turn activates the TGF‐β/Smad signaling pathway and contributes to HCC tumorigenesis and progression [34].

### 3.5 Exploration of Other Related Pathways

Additionally, SNHGs can participate in the occurrence and development of HCC in several ways: by recruiting transcription factors to regulate mRNA transcription, binding directly to mRNAs and proteins, or acting as scaffolds to facilitate complex formation.

Zhang F et al. have shown that SNHG3 recruits the transcription factor E2F1. E2F1 binds to the promoter region of NEIL3, a gene associated with DNA repair. This recruitment activates the transcriptional expression of NEIL3, thereby promoting the proliferation of HCC cells and tumor growth [28].

SNHG5 can directly bind to the mRNA of ribosomal protein RPS3 in HCC cells, facilitating the expression of RPS3 at both the mRNA and protein levels [119]. However, the detailed mechanism through which SNHG5 enhances RPS3 mRNA and protein expression requires further investigation.

Further studies show that SNHG family members in HCC cells directly interact with proteins to increase their stability. For example, SNHG6 in HCC cells directly binds to BOP1, a ribosome biogenesis factor, which strengthens the stability of the BOP1 protein and promotes its expression [74]. Similarly, SNHG17 can bind to the LRPPRC protein, thereby enhancing the stability of the c‐Myc protein [44].

Liu F et al. further discovered that SNHG6 acts as a scaffold facilitating the interaction between FAF2 and mTOR, which in turn promotes cholesterol synthesis and drives the progression of NAFLD to HCC [75].

The aforementioned studies indicate that the SNHG family regulates HCC through multi‐targeted and multi‐modal mechanisms. These mechanisms operate at various levels, including transcription, translation, and protein metabolism, ultimately promoting HCC initiation, proliferation, and progression. The elucidation of these mechanisms provides more potential targets for the subsequent development of HCC‐targeted therapeutic strategies focusing on the SNHG family.

## 4 Role of SNHGs in the Diagnosis and Prognosis of HCC

SNHGs in HCC present a promising avenue for diagnostic and prognostic biomarkers due to their abnormal expression patterns. A comprehensive meta‐analysis has shown that elevated levels of SNHGs are associated with various clinical features of HCC, such as increased tumor dimensions, the presence of multifocal lesions, more advanced histological grading, lymphatic spread, vascular invasion, late‐stage tumors, portal vein thrombosis, elevated AFP concentrations, as well as diminished OS, RFS, and DFS [11]. These findings highlight the potential of measuring SNHGs levels in HCC tissues to improve diagnostic accuracy and patient stratification, which may facilitate earlier therapeutic interventions and better patient outcomes. Nonetheless, the invasive nature of tissue‐based SNHG detection necessitates further research to determine if serum levels of SNHGs are correspondingly elevated in HCC patients, thereby serving as a non‐invasive diagnostic or prognostic biomarker.

Research conducted by Ma X et al. and Gao S et al. has indicated that plasma levels of DANCR and SNHG1 in HCC patients correlate significantly with those in cancer tissues, showing strong associations with critical clinical parameters such as microvascular invasion, invasion of the liver capsule, tumor dimensions, TNM staging, and AFP levels. The diagnostic efficacy of these plasma markers in distinguishing HCC from healthy individuals, as well as from patients with hepatitis B or cirrhosis, appears to be slightly superior to that of AFP [120, 121]. In contrast, certain SNHGs, such as SNHG18, are found to be downregulated in HCC tissues, and their low expression is associated with poor prognosis. Specifically, plasma levels of SNHG18 are significantly reduced in HCC patients when compared to healthy controls, patients with hepatitis B, or those with cirrhosis. Among HCC patients exhibiting AFP levels below 200 ng/mL, SNHG18 shows comparatively high sensitivity and specificity relative to cirrhosis patients and healthy controls, with sensitivity at 75.61% and specificity at 73.49% [122]. These findings imply that serum SNHG levels may serve as critical indicators in the diagnosis and prognosis of HCC. These SNHGs, as non‐invasive biomarkers, hold promise for early diagnosis and prognosis evaluation of HCC. However, most current studies are constrained by limited sample sizes, necessitating large‐scale clinical studies to confirm their sensitivity and specificity.

A meta‐analysis performed by Du SQ et al. revealed that elevated levels of SNHGs are associated with a reduced OS for patients, with a calculated hazard ratio (HR) of 1.697 and a 95% confidence interval (CI) spanning from 1.373 to 2.021. Particularly, SNHG5 exhibited an even higher HR of 4.74, accompanied by a 95% CI that ranges from 1.35 to 6.64, indicating its importance in the prognostic assessment of liver cancer. Furthermore, high expression of SNHGs is also associated with a shorter progression‐free survival (PFS), evidenced by an HR of 1.85 and a 95% CI of 1.25 to 2.73 [12]. This suggests that SNHG could serve as a vital predictive biomarker and a potential therapeutic target for HCC. Nonetheless, further research is necessary to ascertain if serum levels of SNHG expression can be utilized as prognostic indicators for HCC.

## 5 Regulatory Mechanisms of SNHGs

The expression levels of SNHGs are regulated through various mechanisms, including both transcriptional and post‐transcriptional processes. At the transcriptional stage, a variety of transcription factors are instrumental in governing the expression of SNHGs. For instance, transcription factors such as E2F1, c‐Myb, and STAT3 have been documented to interact with the promoters of SNHG1, SNHG10, and DANCR, respectively, thus facilitating their expression [37, 73, 76]. Moreover, epigenetic modifications, including DNA methylation and modifications to histones, significantly influence the transcriptional regulation of SNHGs. Evidence suggests that in glioblastoma, the promoter region of SNHG12 undergoes demethylation, which results in enhanced expression levels [123]. However, it remains to be elucidated whether these regulatory mechanisms are also applicable in HCC and require further investigation.

At the post‐transcriptional stage, the regulation of SNHGs involves various mechanisms including alternative splicing, RNA editing, and RNA stability. A notable instance is SNHG6, which has the capability to produce five distinct transcripts through the process of alternative splicing. While both the SNHG6‐003 and SNHG6‐006 transcripts have been observed to be upregulated in HCC, only the SNHG6‐003 transcript exhibits oncogenic properties, highlighting the significance of RNA alternative splicing in the functionality of SNHG6 [124]. Furthermore, RNA stability plays a pivotal role in modulating the levels of SNHGs. Research has shown that METTL3‐mediated m6A modification promotes the binding of the m6A “reader” protein IGF2BP2 to GAS5, enhances the stability of GAS5, and thereby promotes the expression of GAS5 in HCC cells [18]. However, it remains to be further studied whether other SNHGs in HCC also have similar regulatory mechanisms.

Additionally, RNA editing processes, such as adenosine‐to‐inosine (A‐to‐I) editing, may modify the sequence and structure of SNHGs, potentially impacting their interactions with RNA‐binding proteins. Currently, there is insufficient evidence to suggest that RNA editing has an impact on the expression of SNHGs within HCC, suggesting that further research is warranted in this area.

Compounds such as sorafenib and cholesterol have been demonstrated to elevate the expression levels of SNHG1 and SNHG6 in HCC, and the specific mechanisms need further research [69, 75].

These intricate regulatory pathways collectively influence both the expression levels and functional roles of SNHGs in HCC. A deeper understanding of these mechanisms may clarify the role of SNHGs in HCC development. It also offers new insights for developing SNHG‐targeted therapeutic strategies.

## 6 Potential Therapeutic Targets of SNHGs

By integrating the above findings, it is clear that SNHGs play a crucial role in HCC initiation and progression. This discovery not only highlights their significance in disease mechanisms but also identifies them as promising potential targets for HCC prevention and treatment. Currently, researchers have established a relatively mature system of targeted therapy technologies for lncRNAs, with widely used methods including small interfering RNAs (siRNAs), antisense oligonucleotides (ASOs), the CRISPR/Cas9 gene editing system, and small‐molecule compounds that have gained increasing attention in recent years [125]. Notably, clinical translation studies of other lncRNAs, such as HOTAIR in solid tumor treatment, have achieved phased progress [126]; however, research on SNHG‐targeted therapy remains relatively underdeveloped, with limited reports.

Taking the research by Kim SY's team as an example, the siRNA they designed targeting the lncRNA GAS5 exhibited a significant inhibitory effect on HCC cell proliferation in both in vitro and in vivo experiments [18]. To further improve treatment precision, especially targeting liver tissue, the team encapsulated si‐Gas5 into two liposomal nanoparticles: C12‐SPM and C12‐SPM‐GAL. The experimental results showed that the siRNA mixture loaded with C12‐SPM‐GAL had the most prominent inhibitory effect on tumor growth. This result not only verifies the feasibility of GAS5‐targeted therapy but also provides a reference technical approach for the targeted delivery of lncRNAs such as SNHGs.

ASOs are a class of artificially chemically synthesized DNA sequences (usually 15–25 nucleotides in length). Their mechanism involves forming complementary pairs with target RNAs, which recruits RNase H to trigger RNA degradation. Alternatively, they inhibit transcription by blocking ribosome binding through steric hindrance [127]. Existing studies have confirmed that inhibiting the expression of lncRNAs such as MALAT1, HOTAIR, and NEAT1 using ASO technology can effectively suppress tumor initiation and progression [126, 128, 129]; unfortunately, reports on the use of SNHG‐targeted ASOs for HCC treatment remain extremely scarce, and research in this area urgently requires expansion.

The core mechanism of the CRISPR/Cas9 system in gene editing involves single‐guide RNA forming base complementary pairs with specific genome sequences. This guides the Cas9 nuclease to the target site, where it cleaves DNA downstream of the protospacer adjacent motif [130]. Several research teams have attempted to use this system to knock down or knock out molecules such as SNHG1, SNHG3, SNHG6, and SNHG15. The results showed that these interventions could significantly inhibit the progression of diseases including bladder cancer, neuroblastoma, and leukemia [131, 132, 133, 134]. To date, few studies have reported using the CRISPR/Cas9 system to target SNHGs in HCC. Exploring this approach may offer a new breakthrough for disease treatment.

In addition, research on small‐molecule compounds targeting SNHGs for HCC treatment is also relatively scarce. Existing studies have shown that resveratrol promotes SNHG29 expression and induces its release into exosomes. This process ultimately suppresses HCC occurrence and progression by inhibiting autophagy [70]. However, to ensure research rigor, whether SNHG29 is a specific target of resveratrol's anti‐HCC effects requires further experimental validation. Clarifying this issue will provide a key basis for developing small‐molecule targeted therapies.

Existing studies have initially confirmed that siRNAs, ASOs, and the CRISPR/Cas9 gene editing system have the potential to target SNHGs for HCC treatment. However, to translate this potential into clinical practice, a key challenge remains: how to develop a tissue‐specific delivery system. Such a system should not only deliver gene therapy drugs precisely to HCC lesions but also reduce off‐target effects and improve treatment efficiency. Currently, common gene delivery vectors in the academic community include liposomes and exosomes. As shown in the aforementioned research by Kim SY's team, liposome and nanoliposome vectors can specifically transport siRNAs targeting GAS5 to the liver, significantly inhibiting HCC growth [18]. Regarding exosomes, although studies have shown that exosome‐derived SNHGs regulate HCC development [70, 84], few studies have yet explored using exosomes as carriers to transport SNHG‐targeted siRNAs, ASOs, or CRISPR/Cas9 systems to the liver for HCC therapy. This research gap highlights a promising direction for future studies focused on developing exosome‐based delivery systems for targeted HCC treatment.

## 7 Conclusion

In summary, research on SNHGs in HCC has made significant progress, highlighting their crucial roles and complex mechanisms in HCC development. Multiple studies confirm that aberrant SNHG expression is a hallmark of HCC. It is not merely a byproduct of disease progression but a key driver of various pathophysiological processes. These include disrupted cell proliferation–apoptosis balance, enhanced tumor invasion and migration, cancer stem cell maintenance, dysregulated cell death (such as autophagy, ferroptosis, and pyroptosis), metabolic reprogramming, tumor microenvironment remodeling, and altered therapeutic sensitivity.

## 8 Current Gaps and Future Perspectives

Yet the field faces limitations. Most findings come from in vitro cell assays or tissue analyses, and their in vivo relevance requires validation through animal models and long‐term clinical follow‐ups. Research coverage is narrow: few studies explore SNHGs in emerging cell death types (such as cuproptosis, necroptosis, and disulfidptosis) or in‐depth tumor microenvironment crosstalk involving immune cells, stromal cells, and cytokines. Many novel SNHGs from high‐throughput sequencing also lack functional characterization.

Mechanistically, focus has centered on the classical ceRNA network, while other pathways are understudied, e.g., SNHG‐snoRNA interactions, direct DNA binding for transcription regulation, or impacts on mRNA splicing/protein modifications.

Clinically, SNHG research remains preclinical. While elucidating their mechanisms aids HCC pathogenesis understanding and targeted strategy development, critical issues (in‐human safety, long‐term efficacy, optimized administration) need clinical trial validation. SNHGs also show promise as diagnostic biomarkers due to their tissue‐specific expression and correlation with clinicopathological features. However, they require validation in large multi‐center cohorts and the development of standardized liquid biopsy models before clinical application.

## Author Contributions

Jiajia Luo and Shuangxia Zhang contributed to the conception, data acquisition, and analysis of the research. Jiajia Luo and Zhangxiu Liao drafted and wrote the manuscript.

## Funding

This work was supported in part by grants from the Natural Science Foundation of Guangxi Zhuang Autonomous Region (grant number: 2025GXNSFHA069034) and from the High‐level Talent Program of Youjiang Medical University for Nationalities (grant number: yy2019rcky008).

## Conflicts of Interest

The authors declare no conflicts of interest.

## Data Availability

Data sharing not applicable to this article as no datasets were generated or analysed during the current study.
